# Case report: Experience of a rare case of rebound of the Kasabach-Merritt phenomenon during sirolimus treatment in kaposiform hemangioendothelioma

**DOI:** 10.3389/fped.2022.949950

**Published:** 2022-08-05

**Authors:** Liang Wang, Jing Li, Changhua Wu, Dan Song, Zhuang Liu, Yanli Niu, Jie Zhou, Lei Guo

**Affiliations:** ^1^Department of Vascular Anomalies and Interventional Radiology, Children’s Hospital Affiliated to Shandong University, Jinan, China; ^2^Department of Vascular Anomalies and Interventional Radiology, Jinan Children’s Hospital, Jinan, China; ^3^Shandong Provincial Clinical Research Center for Children’s Health and Disease, Jinan, China

**Keywords:** kaposiform hemangioendothelioma, Kasabach-Merritt phenomenon, embolization, ablation, vincristine, prognosis

## Abstract

Kaposiform hemangioendothelioma (KHE) is a rare vascular neoplasm associated with the Kasabach–Merritt phenomenon (KMP), which is a consumptive coagulopathy with associated potentially life-threatening thrombocytopenia. There are no standardized treatment protocols for the management of KHE with KMP. Moreover, there are limited reports regarding the treatment of cases of rebound. Herein, we describe a rare case of rebound of KHE/KMP, during systemic sirolimus treatment, successfully treated with embolization and vincristine infusion combined with microwave ablation.

## Introduction

Kaposiform hemangioendothelioma (KHE) is a rare type of pediatric vascular tumor, with an incidence of < 0.1:100.000 (1). Moreover, in up to 70% of cases, KHE is complicated by a life-threatening consumptive coagulopathy with severe thrombocytopenia, referred to as the Kasabach-Merritt phenomenon (KMP) (1). For children suffering from the KMP, traditional surgical resection is very difficult, and the mainstay of treatment involves corticotherapy, vincristine infusions, and sclerotic embolization. Furthermore, sirolimus has emerged as a new treatment option because it seems effective for the initial therapy as well as for steroid-resistant KHE, and it is associated with low recurrence rates (1). However, to the best of our knowledge, cases of recurrent KMP during sirolimus treatment have not yet been systematically reported. In this case report, we summarized our experience in the management of a patient who experienced rebound KHE/KMP after embolization during systemic sirolimus administration. Satisfactory results were achieved with a second embolization and vincristine infusion combined with microwave ablation.

## Case report

A 10-year-old girl was referred to our hospital for management of persistent painful purpura on the right chest wall, evolving for 4 months. Three years before this consultation, she was hospitalized in the internal medicine department of our hospital for chest pain. During that period, she underwent closed thoracic drainage because she had developed hydrothorax. Subsequent imaging studies revealed space-occupying lesions in the right chest wall with possible involvement of the ribs and thoracic vertebrae. In addition, there was bilateral hydrothorax, pneumonia with atelectasis in the upper lobe of the right lung, emphysematous changes in the right lung, and a thoracic deformity. Moreover, the hydrothorax was bloody and approximately 200–300 ml of the collection was discharged intermittently one to two times a day. A pathological smear performed revealed several mesenchymal cells, histiocytes, lymphocytes, and neutrophils on a background of red blood cells. She was placed on steroid pulse therapy with dexamethasone (1 mg/kg/d), and she underwent a soft tissue biopsy and transarterial embolization under general anesthesia. During the operation, the tumor-supplying arterial branches including the 8th intercostal artery, internal thoracic artery, and axillary artery were embolized using pingyangmycin iodized oil emulsions. Pathological findings were suggestive of kaposiform hemangioendothelioma. The patient was subsequently treated with oral sirolimus (Dose: 0.5 mg bid), and dexamethasone was changed to equal-dose prednisone (Dose: 17.5 mg po bid) therapy. With this management, her condition effectively improved with purpura disappearing, and control ultrasounds revealed that the right hydrothorax had significantly reduced. The patient was discharged after the removal of the closed thoracic drainage tube.

Then, 8 weeks after her discharge, the patient progressively stops taking the prednisone (by tapering the dose), under her doctor’s instructions. However, she continue taking oral sirolimus (Dose: 0.5 mg bid, with a maintenance target serum level of 5–15 ng/mL) for 3 years, and her condition remained stable. However, ultrasound imaging during the follow-up period revealed that the hydrothorax had persisted. Four months ago, she stopped taking the oral sirolimus for a period of 1 week because she contracted an upper respiratory tract infection. Consequently, the painful skin purpura reappeared. Later on, she resumed taking the regular oral sirolimus (Dose: 0.5 mg bid, on the advice of her doctor); however, her symptoms did not significantly improve, prompting a re-hospitalization. Her laboratory findings a week before her re-hospitalization revealed a platelet count of 75,000/mL (reference range, 100,000–300,000/mL), fibrinogen level of 1.16 g/L (reference range, 2–4 g/L), activated partial thromboplastin time of 39.80 s (reference range, 24–38 s), D-dimer level of 62.61 mg/L (reference range, 0–1 mg/L), and serum level of sirolimus of 13.9 ng/mL.

**FIGURE 1:**
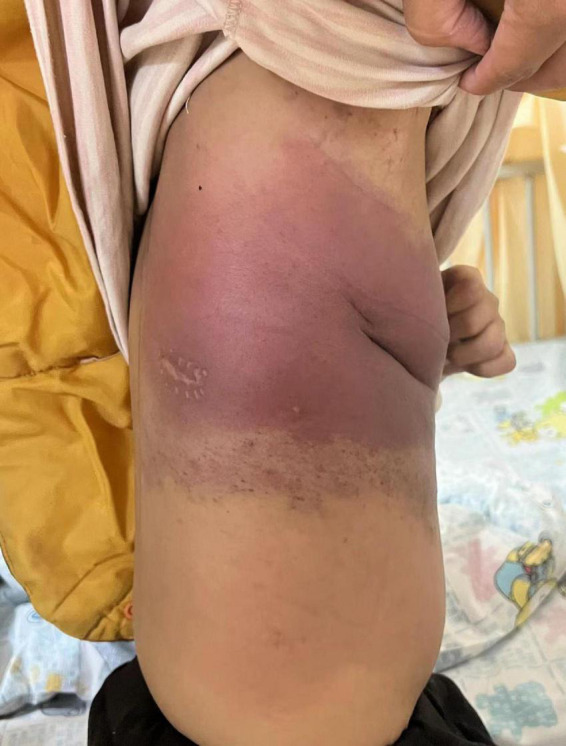
Purpura on the right chest wall.

**FIGURE 2:**
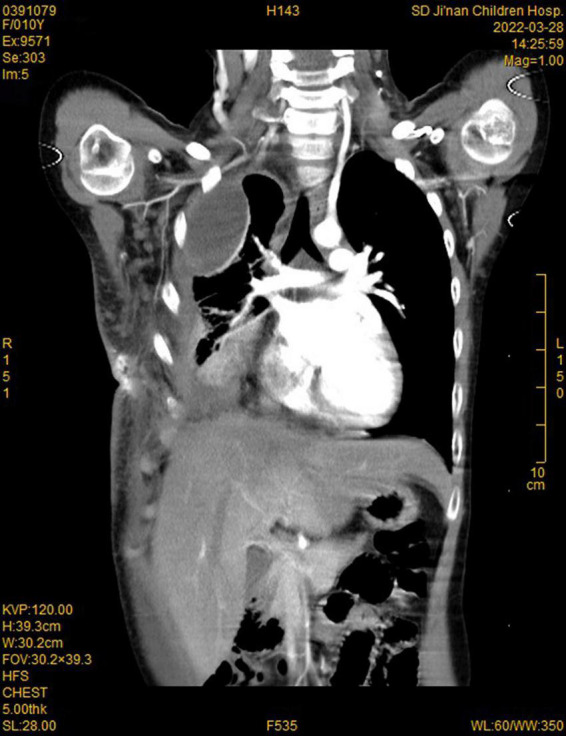
Computed tomography (CT) imaging showed enhancement of chest wall lesions, atelectasis of the right lung, right encapsulated pleural effusion, and pleural thickening.

**FIGURE 3:**
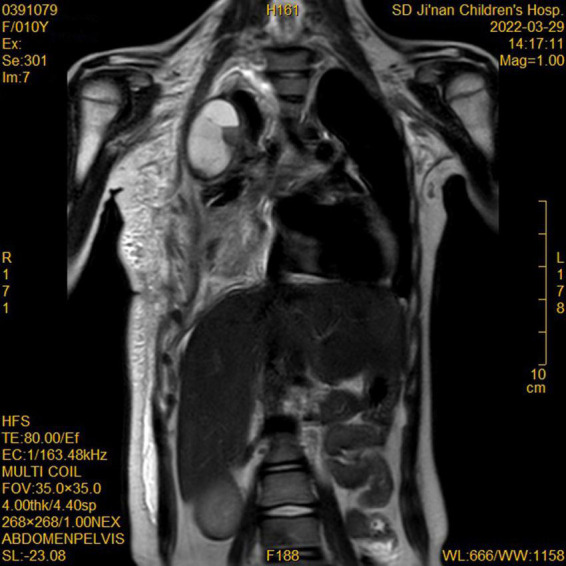
Magnetic resonance imaging (MRI) showed enhancement of chest wall lesions, atelectasis of the right lung, right encapsulated pleural effusion, and pleural thickening.

During this re-hospitalization, a second blood analysis revealed a platelet count of 49,000/mL, fibrinogen level of 1.16 g/L (reference range, 2–4 g/L), activated partial thromboplastin time of 41.7 s (reference range, 24–38 s), and D-dimer level of 63.14 mg/L (reference range, 0–1 mg/L). Physical examination revealed painful purpura with a well-healed local incision scar (of approximately 4 cm in length) on the right chest wall (Figure 1). There were no remarkable findings except for thoracic asymmetry and mild scoliosis. On enhanced chest computed tomography (CT) scan (Figure 2), in addition to the uneven enhancement of the chest wall lesions with unclear margins, pneumonia with atelectasis of part of the right lung, thoracic malformation, right encapsulated pleural effusion, and pleural thickenings were also observed. Similar findings were observed under magnetic resonance imaging (MRI) (Figure 3).

After completing the supplementary analyses, the patient was again treated by sclero-embolization under general anesthesia. Her right lung showed compressive changes under fiberoptic bronchoscopy, and no other abnormalities were observed. During the operation, the tumor-supplying arterial branches, including the right internal thoracic artery, axillary artery, and multiple intercostal arteries, were treated with vincristine (1.2 mg) infusion, and embolization was performed using pingyangmycin iodide oil emulsion and polyvinyl alcohol (PVA) of 300–500 μm-diameter (Supplementary Figures 1, 2). Simultaneously, we performed dynamic real-time ablation of the main lesions using a microwave ablation needle (energy = 15 W) under ultrasound monitoring.

**FIGURE 4:**
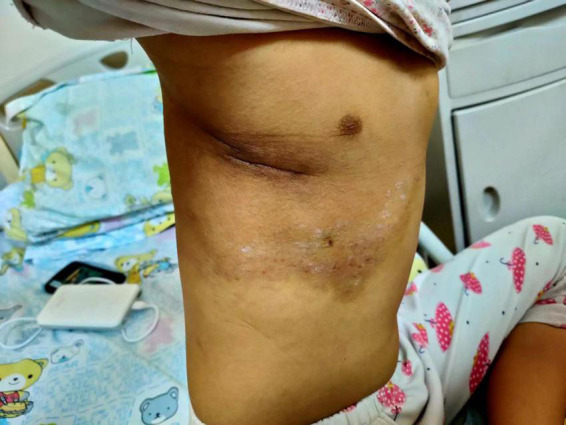
Photo of the child on the 4th postoperative day.

Postoperatively, we administered 5 mg of dexamethasone daily, concomitantly with phenethylamine (a common anticoagulant). No obvious discomforts were observed. However, it should be noted that the skin color of the lesions (Figure 4), platelet count (the comparison is shown in Supplementary Figure 3), and coagulation function significantly improved 4 days after surgery. The dexamethasone was converted to prednisone (Dose: 25 mg po bid) treatment. The patient was discharged on day six post-surgery, and sirolimus (Dose: 0.5 mg bid) was administered orally during the entire treatment period without obvious complications. In addition, cotrimoxazole (Dose: 0.4 g po bid) was also given to prevent the risk associated with sirolimus. Finally, we referred the child to a professional department for the management of the pleural effusion when she became stable.

## Discussion

Kaposiform hemangioendothelioma is a rare vascular neoplasm with locally aggressive characteristics (2). Intrathoracic KHEs are frequently associated with KMP (2, 3). The mortality of KMP is high (10–30%) in KHE patients (1). There are no standardized treatment protocols for the management of KHE with KMP. Currently, the main treatment principles of complicated KHE rely on the remission of the primary tumor, rapid resolution of the life-threatening consumptive coagulopathy, and prevention of recurrence of either condition (1).

The treatment should be aggressive and should involve a combined regimen for patients with concomitant KHE and KMP (2). Corticosteroids and vincristine have been considered the first-line treatments for KHE (4). In the past, vincristine was used in combination with transarterial embolization to treat concomitant KHE and KMP (1). However, sirolimus has recently emerged as a treatment option for complicated vascular anomalies and tumors in children, including KHE with or without KMP (5). Experience so far suggests that systemic sirolimus with or without corticosteroids is needed for most patients with KHE (6). Sirolimus plus steroids are now considered as first-line therapy for the treatment of KHE with KMP (2). In fact, it has been proposed that both corticosteroids and sirolimus have anti-inflammatory effects in a patient with KHE (7). Embolization may bridge the gap between the occurrence of the life-threatening KMP and the effects of systemic treatment, and when combined with the systemic administration of sirolimus in the treatment of KHE, embolization provides a more rapid resolution of the KMP (1). Thermal ablation is a minimally invasive surgical technique that has been widely adopted for the treatment of various solid tumors, and it has gradually become an important tool in the treatment of difficult vascular malformations (8). It can be used as an alternative treatment when other treatment methods are ineffective (9).

The child in this case report has received sirolimus in combination with the corticotherapy was discontinued progressively from the 8th week following the discharge for a period of 3 years after embolization and achieved good results. However, when the KMP recurred, although oral sirolimus did not produce good results this time, a satisfactory result was achieved after combined embolization, vincristine infusion, and microwave ablation. It is worth noting that for children with KHE complicated by pleural effusion, the treatment of the effusion is also crucial, as it is likely one of the factors affecting the long-term prognosis of the patients. Blind drainage during KMP may lead to bleeding and other risks. Therefore, if there are no symptoms of compression, it is recommended to postpone the effusion treatment to when the patient is stable.

## Data availability statement

The original contributions presented in the study are included in the article/Supplementary material, further inquiries can be directed to the corresponding author.

## Ethics statement

The studies involving human participants were reviewed and approved by Ethics Committee of the Children’s Hospital Affiliated with Shandong University. Written informed consent to participate in this study was provided by the participants’ legal guardian/next of kin. Written informed consent was obtained from the minor(s)’ legal guardian/next of kin for the publication of any potentially identifiable images or data included in this article.

## Author contributions

All authors made substantial contributions to conception and design, acquisition of data or analysis and interpretation of data, drafted the manuscript or revised it critically for important intellectual content, and made final approval of the version to be published.

## Supplementary material

Supplementary Figure 1 Angiography showed the supplying artery of the lesion.

Supplementary Figure 2 Angiography showed the supplying artery of the lesion.

Supplementary Figure 3 Platelet count changes during oral sirolimus. **(a)** 10 days after sclero-embolization, purpura disappeared, discharge, sirolimus in combination with the corticotherapy; **(b)** Gradually discontinue prednisone; **(c)** She stopped taking the oral sirolimus for a period of 1 week and the painful skin purpura reappeared, and she resumed taking the regular oral sirolimus; **(d)** Sclero-embolization and microwave ablation, irolimus in combination with the corticotherapy; **(e)** Purpura disappeared; **(f)** Discharge.
