# Supplementary figures and images for: Case report: Experience of a rare case of rebound of the Kasabach-Merritt phenomenon during sirolimus treatment in kaposiform hemangioendothelioma
Source: Front Pediatr. 2022 Aug 5;10:949950. doi: 10.3389/fped.2022.949950 (PMC9391052; doi:10.3389/fped.2022.949950)

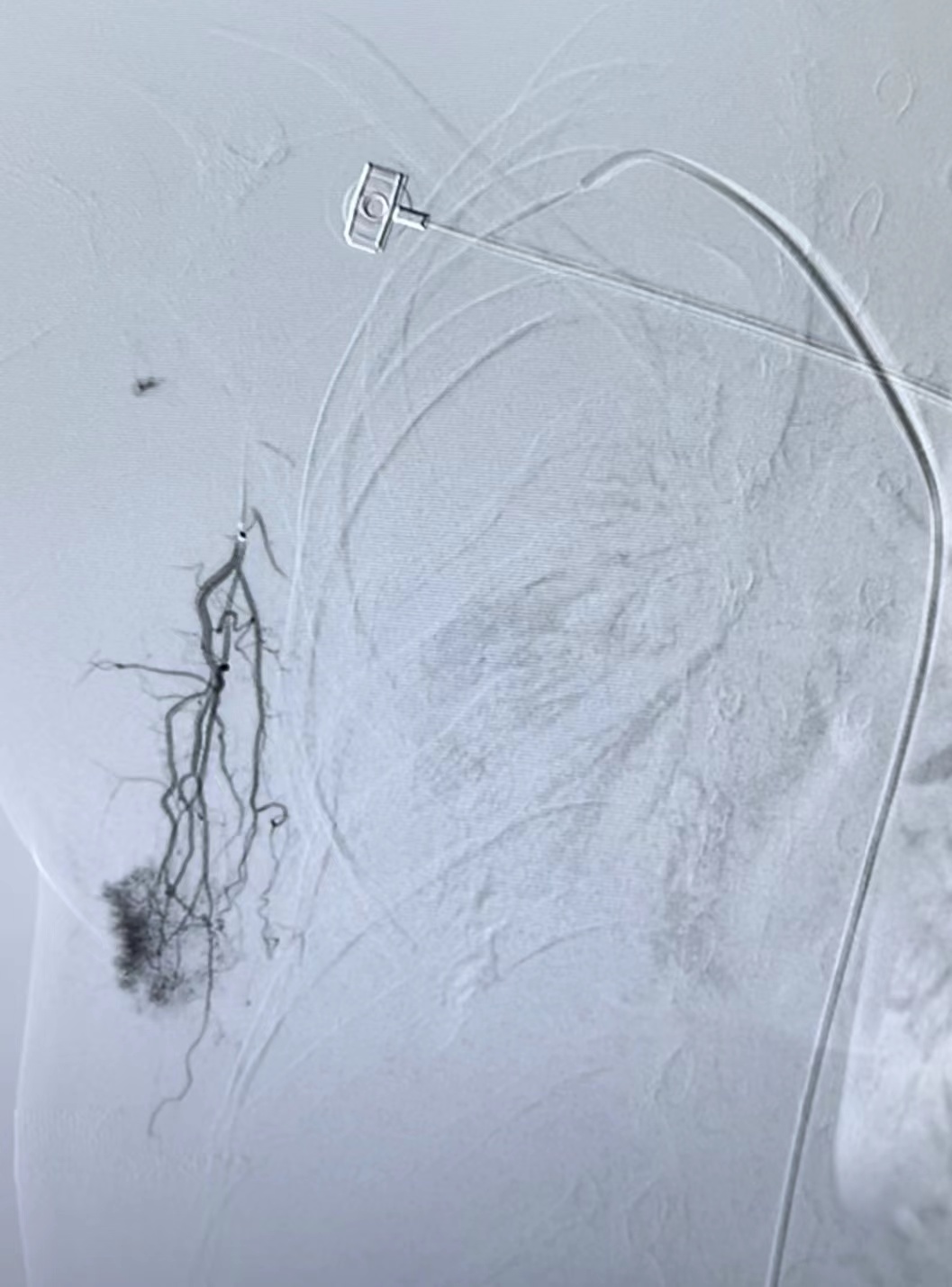

Supplement: Supplementary Figure 1 — Angiography showed the supplying artery of the lesion. [file Image_1.JPEG]

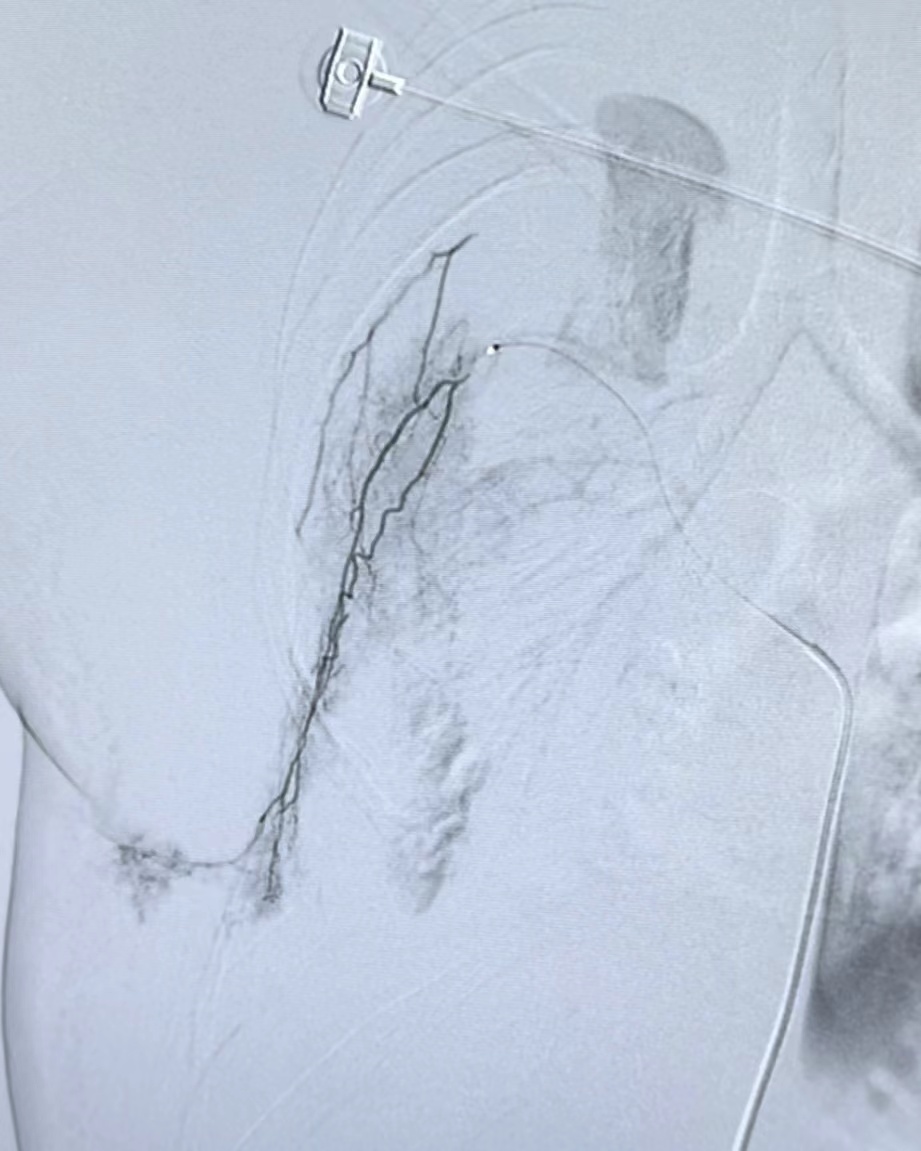

Supplement: Supplementary Figure 2 — Angiography showed the supplying artery of the lesion. [file Image_2.JPEG]

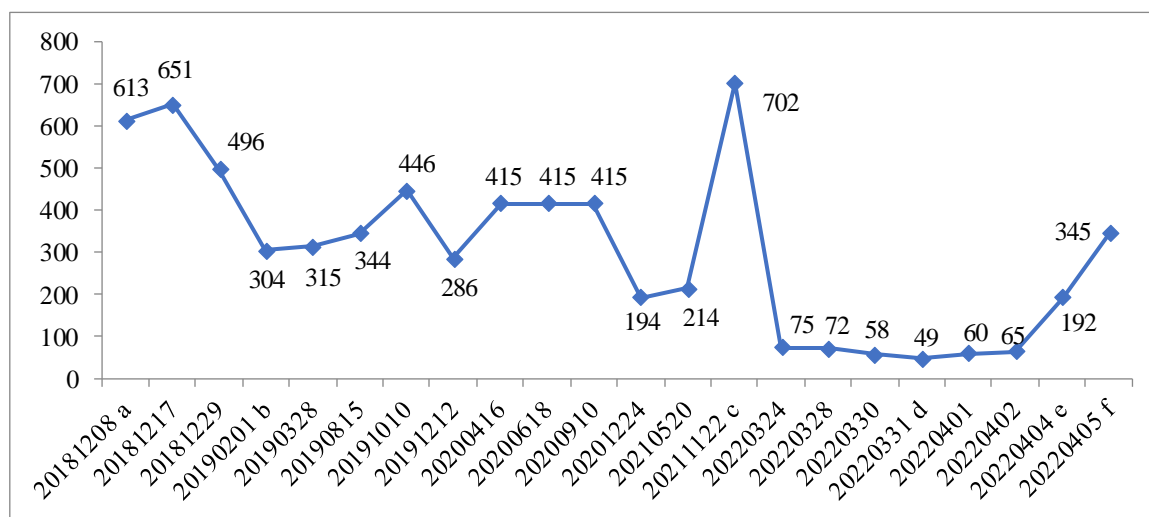

Supplement: Supplementary Figure 3 — Platelet count changes during oral sirolimus. (a) 10 days after sclero-embolization, purpura disappeared, discharge, sirolimus in combination with the corticotherapy; (b) Gradually discontinue prednisone; (c) She stopped taking the oral sirolimus for a period of 1 week and the painful skin purpura reappeared, and she resumed taking the regular oral sirolimus; (d) Sclero-embolization and microwave ablation, irolimus in combination with the corticotherapy; (e) Purpura disappeared; (f) Discharge. [file Image_3.pdf]
